# Evidence for biomolecular condensates formed by the *Escherichia coli* MatP protein in spatiotemporal regulation of the bacterial cell division cycle

**DOI:** 10.1016/j.ijbiomac.2025.142691

**Published:** 2025-03-31

**Authors:** Inés Barros-Medina, Miguel Ángel Robles-Ramos, Marta Sobrinos-Sanguino, Juan Román Luque-Ortega, Carlos Alfonso, William Margolin, Germán Rivas, Begoña Monterroso, Silvia Zorrilla

**Affiliations:** a Department of Cellular and Molecular Biosciences, Centro de Investigaciones Biológicas Margarita Salas, Consejo Superior de Investigaciones Científicas (CSIC), 28040 Madrid, Spain; b Molecular Interactions Facility, Centro de Investigaciones Biológicas Margarita Salas, Consejo Superior de Investigaciones Científicas (CSIC), 28040 Madrid, Spain; c Department of Microbiology and Molecular Genetics, McGovern Medical School, UTHealth-Houston, Houston, TX 77030, USA; d Department of Crystallography and Structural Biology, Instituto de Química Física Blas Cabrera, Consejo Superior de Investigaciones Científicas (CSIC), 28006 Madrid, Spain

**Keywords:** Macromolecular interactions, Biophysical techniques, Phase separation

## Abstract

An increasing number of proteins involved in bacterial cell cycle events have been recently shown to form biomolecular condensates important for their functions that may play a role in development of antibiotic-tolerant persister cells. Here we report that the *E. coli* chromosomal Ter macrodomain organizer MatP, a division site selection protein coordinating chromosome segregation with cell division, formed biomolecular condensates in crowding cytomimetic systems preferentially localized at the membrane of microfluidics droplets. Condensates were antagonized and partially dislodged from the membrane by DNA sequences recognized by MatP (*matS*), which partitioned into them. FtsZ, a core component of the division machinery previously described to phase-separate, unexpectedly enhanced MatP condensation. Our biophysical analyses uncovered direct interaction between both proteins, disrupted by *matS.* This may have potential implications for midcell FtsZ ring positioning by the Ter-linkage, which comprises MatP and two other proteins bridging the canonical MatP-FtsZ interaction. FtsZ/MatP condensates interconverted with GTP-triggered bundles, suggesting that local fluctuations of GTP concentrations may regulate FtsZ/MatP phase separation. Consistent with discrete MatP foci previously reported in cells, phase separation might influence MatP-dependent chromosome organization, spatiotemporal coordination of cytokinesis and DNA segregation, which is potentially relevant for cell entry into dormant states that can resist antibiotic treatments.

## 1. Introduction

Biomolecular condensation through phase separation is established as a mechanism to regulate cellular function and pathology in eukaryotes, their functions spanning different length scales [1–4]. Although its identification in bacteria is more recent, increasing evidence supports both its key role in providing a mechanism for compartmentalization of these cytoplasms lacking membrane-bound organelles, and additional levels of regulation for multiple cellular processes [5–11]. Biomolecular condensates are dynamic assemblies of a single or various scaffold biomolecules in which others, termed clients, selectively concentrate, providing spatial control to reactions [12,13]. These condensates reversibly form under a narrow range of conditions [14], being favored by multivalency and macromolecular crowding, through volume exclusion or other nonspecific weak interactions [1,15]. Proteins from *Escherichia coli*, *Bacillus subtilis* or *Caulobacter crescentus* involved in chromosome replication, segregation and cell division assemble biomolecular condensates relevant for their function, in cytomimetic systems mimicking the crowded bacterial cytoplasm or in vivo ([6] and references therein). In many instances, biomolecular condensation of proteins, often regulated by nucleic acids, enhances cell fitness and survival in response to different kinds of stresses. In the particular case of bacteria, condensates conferring resistance to starvation, heat shock, phage infection, oxidative stress, or treatments with antibiotics have been identified [6,11,16], suggesting that these structures may represent attractive new targets for the fight against antimicrobial resistance [11,17]. Biomolecular condensates can also be exploited for synthetic biology applications, including the design of artificial cells with compartments that add an additional level of control for complex reactions [18–21]. Taking advantage of the extensive research on the factors driving phase-separation, it has been recently demonstrated that functional biomolecular condensates can be engineered to achieve targeted cellular functions in mammalian or bacterial cells [20].

MatP is a specific DNA binding protein targeting *matS* sites, which are repeated over 20 times at the Ter region of the chromosome, playing a pivotal role in its organization and compaction [22,23]. In vivo, MatP accumulates at precise locations in the cell, forming discrete foci [22,24–28]. Besides the specific recognition of DNA, the modular structure of MatP [24] comprises multiple regions of interaction with itself (leading to dimerization), with other proteins and with lipid membranes. Together with ZapA and ZapB, MatP forms part of the Ter-linkage that, in coordination with the Min system and SlmA-mediated nucleoid occlusion, positions the FtsZ cell division ring at midcell [29]. Within the Ter-linkage, MatP directly interacts through its C-terminal region with ZapB [25], and ZapA links ZapB with FtsZ [30]. Aside from this canonical sequence of interactions, it has been suggested that ZapB may directly bind FtsZ as well [31,32]. Through the Ter-linkage, MatP is a key player in the coordination of chromosome segregation with cell division [25,33,34]. In addition, MatP interacts with the structural maintenance of chromosomes complex MukBEF, contributing to normal chromosome organization and timely unlinking of replicated chromosomes, allowing their segregation [35,36]. Thus, MatP delays sister Ter segregation by releasing MukBEF from the Ter region, which limits the availability at this location of Topoisomerase IV that normally removes chromosomal catenanes. MatP binds lipid membranes, which may play a role in the regulation of its function through modulation of its localization and recognition of other ligands [26].

One of the bacterial proteins that undergo phase separation is FtsZ, which forms biomolecular condensates strongly promoted by macromolecular crowding [37] and through interaction with the nucleoprotein complexes of SlmA [38]. FtsZ condensates and polymers interconvert in response to GTP addition and depletion after GTP hydrolysis by FtsZ, and modulation of this switch by agonists/antagonists has been proposed to contribute to the exquisite regulation of FtsZ ring assembly under normal growth conditions [39]. Additionally, since they sequester FtsZ together with a strong polymerization antagonist (SlmA), these cell division condensates could be among those assembled in dormant cell states tolerant to antibiotics, in which GTP levels are reduced and vital processes like division shut down [40,41]. Consistent with this, aggresomes enriched in FtsZ have been identified in persister cells surviving antibiotic treatment that dissociate when the cells resume growth [42].

Here, by reconstitution in crowding conditions and in cytomimetic systems generated by microfluidics, we show that MatP forms dynamic reversible biomolecular condensates, as revealed by confocal microscopy and turbidity analyses. Condensation was strongly enhanced by FtsZ, with the two proteins assembling heterotypic condensates under conditions in which they do not significantly phase separate on their own. An orthogonal approach combining different biophysical techniques (fluorescence correlation spectroscopy, FCS; fluorescence anisotropy and sedimentation velocity, SV) uncovered direct interaction between MatP and both oligomers and GTP-triggered polymers of FtsZ. We also investigated the impact of *matS* sequences on the assembly of MatP biomolecular condensates and on the interactions with FtsZ. The formation of *matS-*responsive MatP biomolecular condensates, sensitive to GTP when coassembled with FtsZ, could be relevant for the modulation of MatP spatial distribution and interaction with protein partners, to orchestrate its role in FtsZ ring positioning and chromosome organization/segregation. MatP condensates might also contribute to mechanisms determining bacterial resistance to stress conditions, as sequestration of MatP and FtsZ would promote cell cycle arrest, facilitating entrance into a stress-resistant quiescent state. Condensates of cell division proteins such as those identified and reconstituted in microfluidic droplets herein can be exploited in synthetic biology applications. For example, artificial cells can be designed with distinct functional compartments that could more faithfully replicate life-like properties and behaviors.

## 2. Results

### 2.1. MatP forms biomolecular condensates in crowded cytomimetic systems

**Fig. 1.**
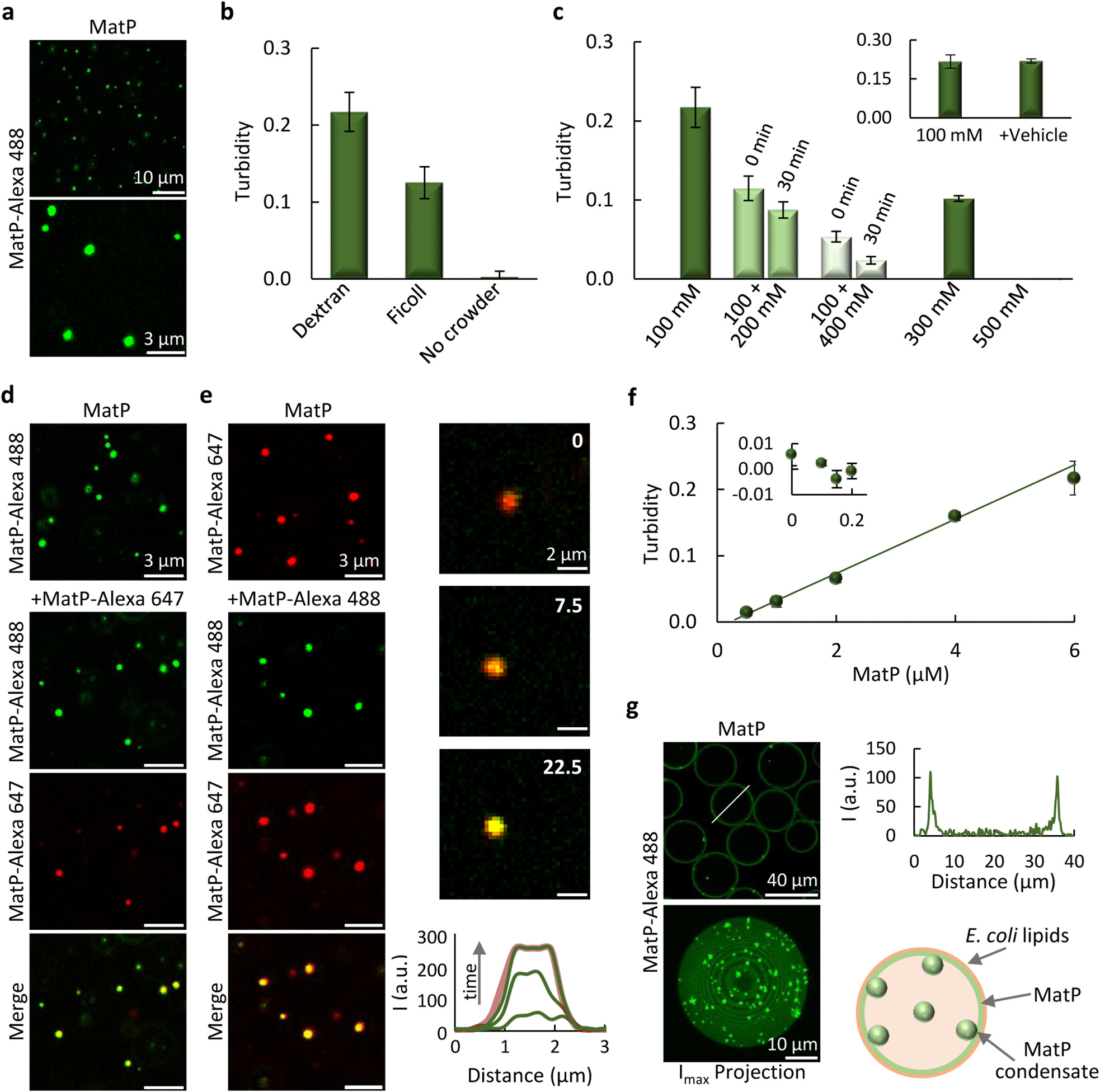
MatP forms biomolecular condensates in crowding conditions. (a) Confocal microscopy images of MatP condensates. (b) Turbidity of MatP samples in dextran (*n* > 5), Ficoll (*n* = 3) or in diluted solution (*n* = 4). (c) MatP turbidity decrease with the sequential addition of KCl, from 100 (*n* > 5) to 300 and 500 mM monitored at the specified times (*n* = 3), showing reversibility of MatP condensates. Samples directly prepared at these KCl concentrations are also shown (*n* = 3). Inset, control of dilution effects by adding vehicle (*n* = 3). (d, e) Images showing incorporation of externally added MatP into MatP condensates, indicating that they are dynamic. Right column in (e) shows the stepwise diffusion of added MatP-Alexa 488 into the condensates at the specified times in seconds (time 0, beginning of the visualization of this particular condensate) with the corresponding intensity profiles below. The profile in the red channel at 22.5 s is shown as reference. (f) Turbidity increase with MatP concentration (*n* = 3 except for 6 μM, *n* > 5). Line corresponds to a linear model fit, rendering the *c*_sat_ in the main text. Inset shows data below *c*_sat_ (*n* ≥ 3). Error bars are within the data symbols in most cases. (g) Left, images of equatorial section (top) and maximum intensity projection (bottom) of microdroplets containing MatP condensates, located mostly at the lipid membrane. Right, intensity profile corresponding to the line drawn on the image and illustration of condensates distribution. MatP concentration was 5 μM (a, d, e, g) or 6 μM (b, c). Labeled MatP was at 1 μM, except in (d–e) that was at 0.5 μM. Errors are SD. All experiments were performed under the *MatP-crowding conditions* (see Materials and methods), substituting Ficoll for dextran or without crowder in (b) as stated.

Confocal images of purified MatP, with Alexa Fluor 488 labeled MatP (MatP-Alexa 488) as tracer, in solutions with the crowding agent dextran revealed the presence of relatively small round structures compatible with biomolecular condensates (Figs. 1a and S1a). They were also observed in the presence of Ficoll (Fig. S1b), but not without crowder (Fig. S1c). This was consistent with the significant turbidity of MatP solutions with crowders, and the negligible values in dilute solution (Fig. 1b). To get some insight into the nature of these MatP structures, we analyzed their response to changes in ionic strength. We found that increasing salt disfavored MatP condensation, reflected in gradually lower turbidity and reduction in the abundance of condensates in the images (Figs. 1c and S2a), suggesting a role of electrostatic interactions in the assembly of MatP condensates.

We also asked whether these structures were dynamic and reversible, features inherent to biomolecular condensates. Condensation was reversible, as addition of supplementary KCl to 100 mM solutions containing the assemblies reduced their amount (Fig. S2b) and turbidity, which approximately matched that of samples directly prepared at that final salt concentrations (Fig. 1c). A modest time-dependent dissociation was observed (Fig. 1c), consistent with the slightly higher abundance of condensates in images acquired at short times after KCl addition compared with those of condensates directly assembled at the final KCl (cf. Figs. S2a and b). Incorporation of externally added MatP, labeled with either Alexa Fluor 647 (MatP-Alexa 647) or with Alexa Fluor 488, into condensates containing MatP labeled with a spectrally different dye confirmed they were dynamic (Figs. 1d, e and S2c). MatP condensation followed the characteristic behavior associated with protein phase separation in which condensates emerge above a saturation concentration (*c*_*sat*_) [2,15]. A *c*_*sat*_ of 0.2 ± 0.1 μM was retrieved from the linear increase in turbidity with MatP concentration (Fig. 1f), below which negligible values consistent with a single phase were obtained (Fig. 1f, inset). Collectively, these experiments indicate that MatP forms dynamic reversible biomolecular condensates driven by crowding.

We further studied the properties of MatP condensates by reconstitution in microfluidics droplets containing dextran as crowder and stabilized by a monolayer of the lipid ternary mixture found in the *E. coli* inner membrane. Maximum intensity projections clearly showed defined condensates that, according to the distribution in the equatorial image, were located mainly within the droplets lipid surface (Figs. 1g and S3). These experiments proved that MatP forms condensates in confined cell-like systems displaying crowding and a lipid membrane, with a clear preference for the latter, consistent with its known tendency to interact with membranes [26].

### 2.2. matS DNA accumulates in MatP condensates, generally disfavoring their formation

**Fig. 2.**
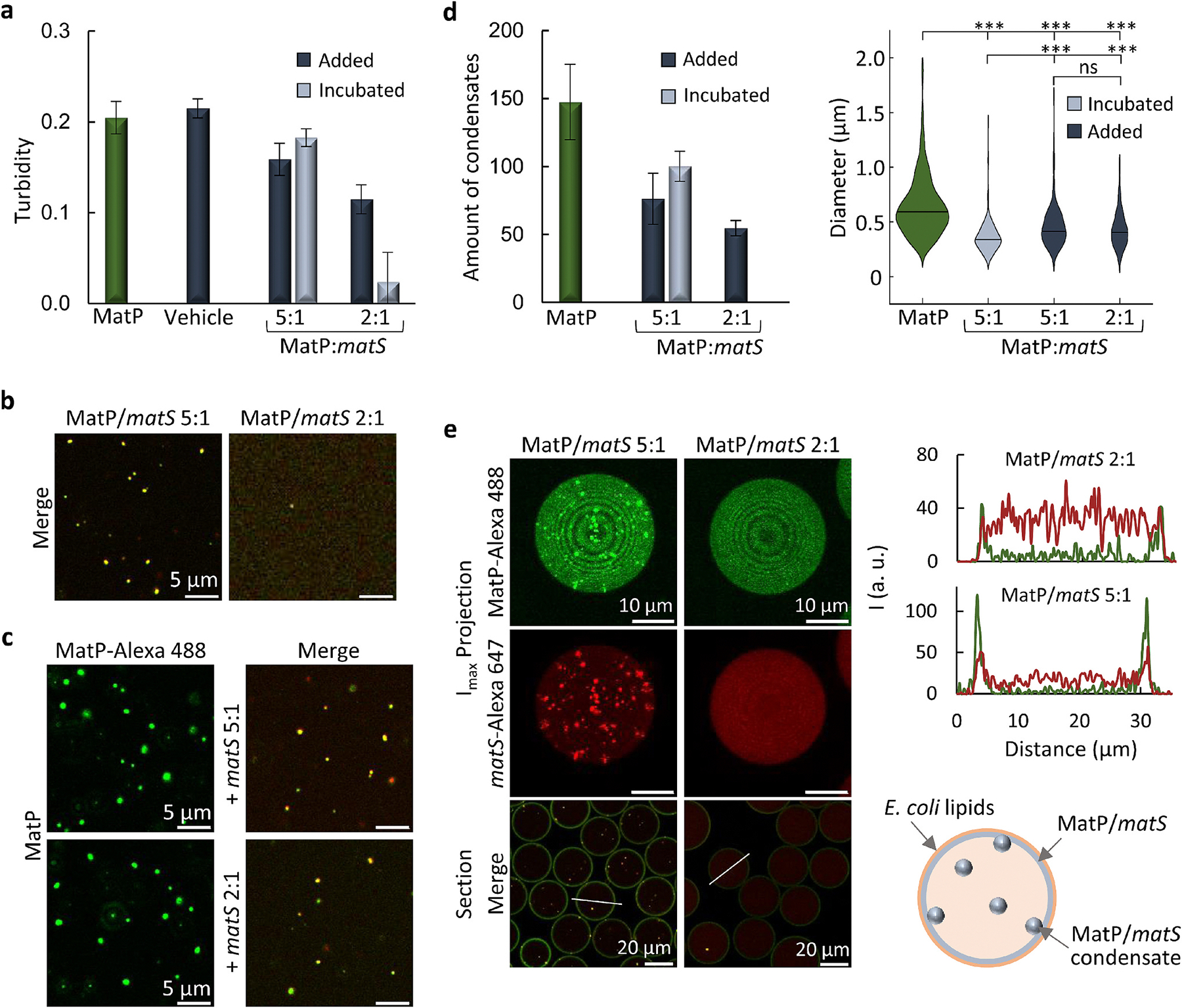
Concentration-dependent modulation of MatP condensates by *matS*. (a) MatP turbidity without (*n* = 5) and with *matS,* added on preformed (30 min-incubated) MatP condensates (data taken 10 min after addition, *n* ≥ 6), or incubated with MatP and the crowder for 30 min (*n* = 3). Dilution effects were ruled out by adding vehicle (*n* = 3, 10 min). Errors are SD. (b, c) Confocal images showing concentration-dependent *matS* inhibition of MatP condensation when incubated before condensates assembly (b) or abundance reduction after addition to preformed condensates (c). MatP and *matS* colocalize in the remaining condensates. Images of the independent green and red channels in Fig. S4. (d) Average number of condensates per image without and with *matS* added on preformed condensates or incubated with MatP from the beginning, at the specified ratios. Nine (without *matS*) or six (with *matS*) independent confocal images (82 × 82 μm fields) were analyzed. On the right, diameters of the condensates shown as violin plots (*n* = 542, 269, 253 or 170 for MatP, MatP:*matS* 5:1 (incubated), MatP:*matS* 5:1 (added), MatP:*matS* 2:1 (added)).***p ≤ 0.001, ns, not significant, by ANOVA on ranks test (Kruskal-Wallis) followed by Dunn’s test. (e) Maximum intensity projections of the independent channels and merge equatorial sections of microdroplets containing MatP incubated with *matS*. Intensity profiles corresponding to the line drawn on the images and a scheme of condensate distribution at the lipid membrane and lumen, at 5:1 MatP:*matS* molar ratio, are shown on the right. Images of the independent green and red channels of the equatorial sections in Fig. S5. MatP concentrations were 6 μM (a) or 5 μM (b, c, d and e) and *matS* concentrations corresponded to the specified MatP:*matS* molar ratios. Labeled components were at 1 μM. All experiments were performed under the *MatP-crowding conditions* (see Materials and methods).

Since nucleic acids are among the strongest modulators of protein phase separation [1], we tested whether the *matS* DNA sequences specifically recognized by MatP influence its condensation. Interestingly, double-stranded (ds) oligonucleotides containing a single *matS* site (hereafter *matS*) disfavored MatP phase separation, to a higher or lower extent depending on the protein:DNA ratio and on whether *matS* was added to the condensates or incubated with MatP during sample preparation (Fig. 2a). Thus, incubation with *matS* at 5:1 MatP:*matS* molar ratio only slightly decreased turbidity regarding MatP alone, while at 2:1 ratio turbidity substantially lowered, indicating that *matS* prevented significant MatP condensation at this higher concentration (Figs. 2a and S4a). Notably, addition of *matS* into preassembled MatP condensates had a lower impact, reflected in a modest turbidity decrease at 5:1 MatP: *matS* ratio, somewhat larger at 2:1 ratio (Figs. 2a and S4b). Consistent with these results, confocal analysis showed lower amounts of condensates when MatP was incubated with *matS* at 5:1 molar ratio, virtually disappearing at 2:1 ratio (Figs. 2b and S4c), and a significant decrease in the amount of condensates when *matS* was added to them instead, at both ratios tested (Figs. 2c, d, S1b and S4d). Measurements of their dimensions in the presence of *matS* (Figs. 2d and S4e) showed a slight decrease with respect to that obtained in the absence of the specific DNA (Fig. 2d). Interestingly, colocalization of Alexa Fluor 647 labeled *matS* (*matS*-Alexa 647) with MatP-Alexa 488 (Fig. 2b, c) evidenced the partitioning of *matS* into the condensates and suggested that MatP within them was still able to bind *matS*. Therefore, specific DNA reduced MatP condensation in a concentration-dependent manner, more efficiently when MatP/*matS* complexes were formed before MatP phase separation, and incorporated into the remaining condensates.

MatP was then co-encapsulated with *matS* inside the above described microfluidics droplets (Figs. 2e and S5a, b), using two aqueous streams with premixed MatP and *matS*. At 5:1 MatP:*matS* molar ratio, *matS* did not have a significant impact on the amount of condensates regarding that observed in its absence (Fig. S5c), whereas at 2:1 ratio no condensates were detected (Fig. 2e). The specific DNA colocalized with the MatP condensates that, still showing a preference for the membrane, increased their abundance in the lumen (Fig. S5d). Intensity profiles showed accumulation of MatP at the membrane at both MatP:*matS* ratios, while *matS* was distributed between lumen and membrane (Figs. 2e and S5a). These experiments showed that, when at sufficiently high concentration, *matS* hindered MatP phase separation in cytomimetic systems. At lower concentrations, *matS* DNA fragments partitioned into the condensates, reducing their tendency to assemble at the membrane of the microdroplets.

To evaluate the specificity of the effects of *matS* sequences, we tested ds-oligonucleotides of similar length but lacking this specific site (Fig. S6). A modest reduction with respect to MatP turbidity was found when the DNA and the protein were incubated at 1:1 molar ratio prior to condensation, whereas the signal was basically unperturbed by DNA addition on preassembled condensates even after 30 min (Figs. S6a and S4b). Accordingly, confocal images showed no significant variation in the condensates abundance or size under most conditions assayed (Fig. S6b, c), with irregular structures appearing upon incubation at 1:1 MatP: DNA molar ratio (Fig. S6c). These sequences devoid of *matS* sites still partitioned into the condensates, probably due to nonspecific interactions with MatP favored by the high protein concentration. Specificity was further tested using a single-stranded *matS* sequence (ss-*matS*) instead of the double-stranded *matS* sequence specifically recognized by MatP. The resulting condensates exhibited colocalization of both the protein and DNA, and were similar in shape and abundance to those formed by MatP alone (Fig. S6d). These results show that, unlike the specific *matS* sequences, nonspecific DNA or ss-*matS* did not significantly disrupt MatP condensates, even when they accumulated inside them.

### 2.3. MatP forms heterotypic condensates with FtsZ

**Fig. 3.**
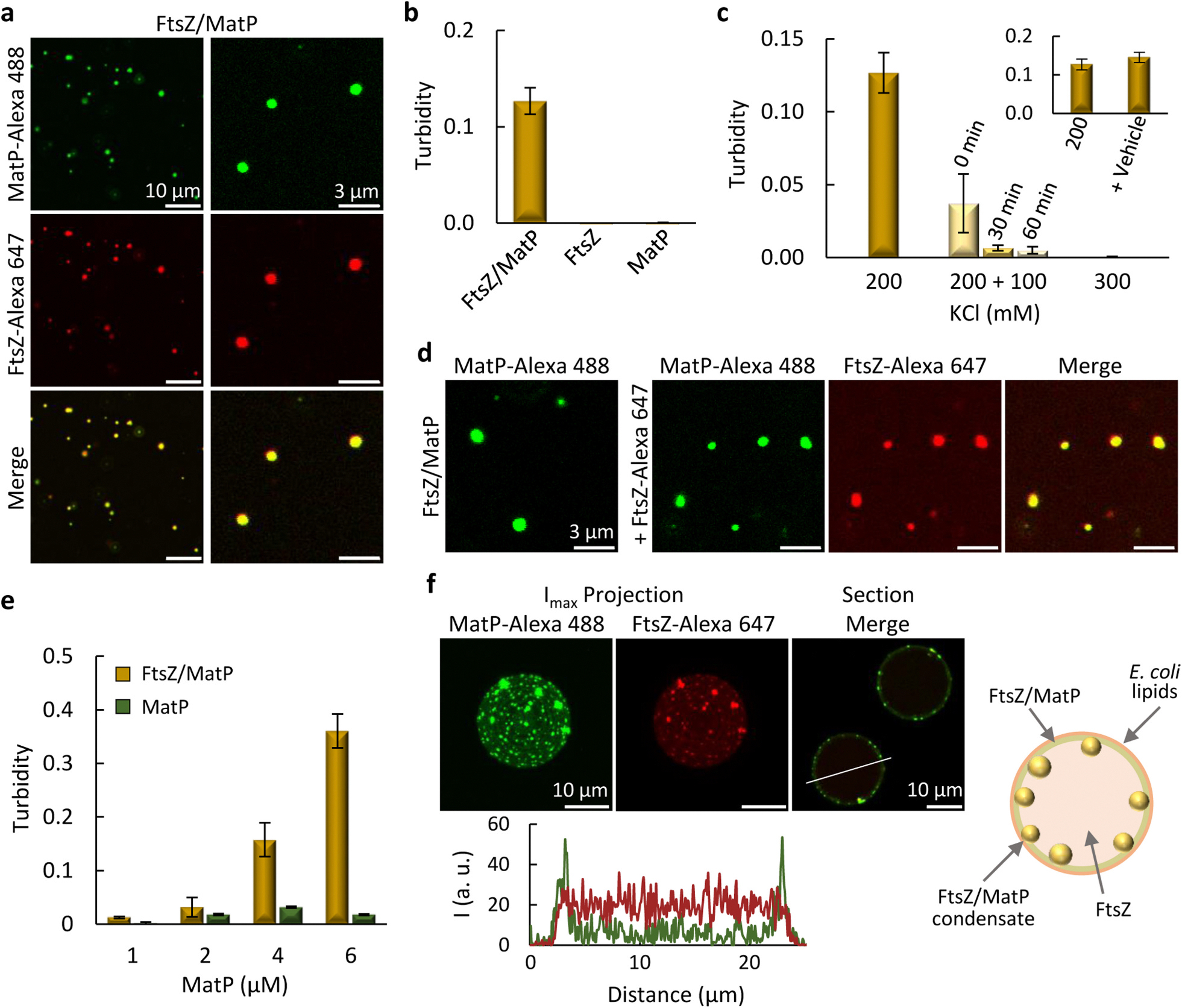
MatP forms heterotypic condensates with FtsZ. (a) Confocal images showing FtsZ/MatP condensates. (b) Turbidity of FtsZ/MatP condensates and lack of signal for only FtsZ or MatP, indicative of no condensation under these conditions. (c) Time-dependent turbidity decrease of FtsZ/MatP condensates shifted from 200 to 300 mM KCl, indicating reversibility. Turbidity of samples prepared at 300 mM KCl is also shown. Inset reflects the absence of dilution effects, by adding vehicle. (d) FtsZ/MatP condensates incorporate externally-added FtsZ, stating they are dynamic. (e) Turbidity increase of FtsZ/MatP condensates with protein concentration, keeping a 2:1 (FtsZ:MatP) molar ratio. Turbidity of MatP alone is also shown. (f) Maximum intensity projections of the independent channels and merge equatorial sections of microdroplets containing condensates formed by mixing FtsZ and MatP at the droplet formation junction. On the right, schematic illustration of condensates distribution mainly at the lipid membrane and, below, intensity profiles of the green and red channels along the line depicted on the image. Concentrations were 5 μM FtsZ, 3 μM MatP and 1 μM labeled components, unless otherwise stated. Data in (b, c) are the average of 3 independent experiments, except for FtsZ/MatP at 200 mM KCl (*n* > 5); in (e) *n* = 4 and *n* = 3 for FtsZ/MatP and MatP, respectively, except when MatP was at 6 μM, *n* > 5. Errors are SD. All experiments were performed under the *FtsZ/MatP-crowding conditions* (see Materials and methods), except for salt variations (c).

FtsZ has been shown to assemble heterotypic condensates in the absence of GTP through interaction with other bacterial division proteins, acting as scaffolds [38] for additional client proteins [39]. Therefore, even though FtsZ is thought to interact with MatP only indirectly [30], we asked whether there could be an interplay between FtsZ and the MatP condensates. Interestingly, FtsZ notably increased MatP solutions turbidity (Fig. S7a) and confocal imaging showed numerous structures (Fig. S7b, left) where the two proteins mostly colocalized, appearing more irregular than the typical round biomolecular condensates, in the conditions used to study the above described homotypic MatP condensates (50 mM Tris-HCl, pH 7.5, 200 g/L dextran, 100 mM KCl, 5 mM MgCl_2_, *MatP-crowding conditions*). Since biomolecular condensates usually form under a narrow range of conditions, and deviation from them often results in either absence of condensation or formation of other types of structures, we explored FtsZ/MatP mixtures at different KCl, MgCl_2_ and crowder concentrations. Reducing dextran from 200 to 150 g/L (Fig. S7b) and, even more, increasing KCl to 200 mM, rendered relatively abundant structures, mostly round and hence compatible with biomolecular condensates, at both 1 and 5 mM MgCl_2_ (Figs. 3a and S8).

After this screening, we chose to characterize FtsZ/MatP condensates at 150 g/L dextran, 200 mM KCl and 1 mM MgCl_2_ (*FtsZ/MatP-crowding conditions*, Fig. 3a). Under these conditions, no condensates were observed with FtsZ alone, in good agreement with previous reports [37], while MatP condensation was subtle (Fig. S9), and significant turbidity indicative of condensation was obtained only when both proteins were present (Fig. 3b). Increasing the KCl content decreased the turbidity to negligible values (Fig. 3c), and drastically reduced the number of condensates in the images (Fig. S10a). FtsZ/MatP condensates were also formed in the presence of Ficoll (Fig. S11a), while they did not assemble without a crowding agent (Fig. S11b). We next tested whether the FtsZ/MatP structures were dynamic and reversible. Addition of KCl to already assembled condensates reduced their abundance in the images and the turbidity to values similar to those of condensates formed at the final KCl concentration (Figs. 3c and S10b), indicating that they were reversible. FtsZ/MatP condensates assembled with MatP-Alexa 488 as tracer readily incorporated externally added FtsZ labeled with Alexa Fluor 647 (FtsZ-Alexa 647), proving they were dynamic (Fig. 3d), as further confirmed by adding MatP-Alexa 488 on preassembled FtsZ-Alexa 647 labeled condensates (Fig. S10c). Turbidity measurements at a fixed 2:1 FtsZ:MatP ratio, while increasing the overall concentration, rendered negligible values at low concentrations (as for MatP solutions without FtsZ at all concentrations tested), followed by the typical concentration-dependent increase compatible with phase separation at higher concentrations (Fig. 3e). Altogether, these experiments evidenced FtsZ/MatP heterotypic biomolecular condensation favored by crowding and electrostatic forces. Here, both proteins behave as scaffolds, as they both are required for condensation under conditions precluding their individual phase separation, observed under other conditions (see above and [37]).

FtsZ/MatP condensates were also analyzed in the microfluidics droplets displaying crowding, confinement and a lipid membrane. To this end, MatP and FtsZ, with MatP-Alexa 488 and FtsZ-Alexa 647 as tracers, were jointly encapsulated with dextran as crowder. Numerous condensates in which both proteins colocalized were observed almost exclusively at the membrane (Figs. 3f and S12a). As in the absence of MatP, FtsZ outside the condensates was found homogeneously distributed in the lumen, and also at the membrane (cf. Figs. 3f and S12b, and intensity profiles therein), whereas MatP was mostly at the membrane, either in the form of condensates or as a continuum, with low intensity detected in the lumen. Analogous results were obtained when encapsulating the preformed FtsZ/MatP condensates or when solutions containing each of the proteins were mixed at the droplet formation junction, to trigger their assembly just before encapsulation (cf. Figs. 3f and S12c). These experiments showed that FtsZ and MatP form condensates in confined and crowded cell-like systems, with high preference for the lipid membrane.

### 2.4. Negative regulation of condensation by matS is sustained for FtsZ/MatP condensates

**Fig. 4.**
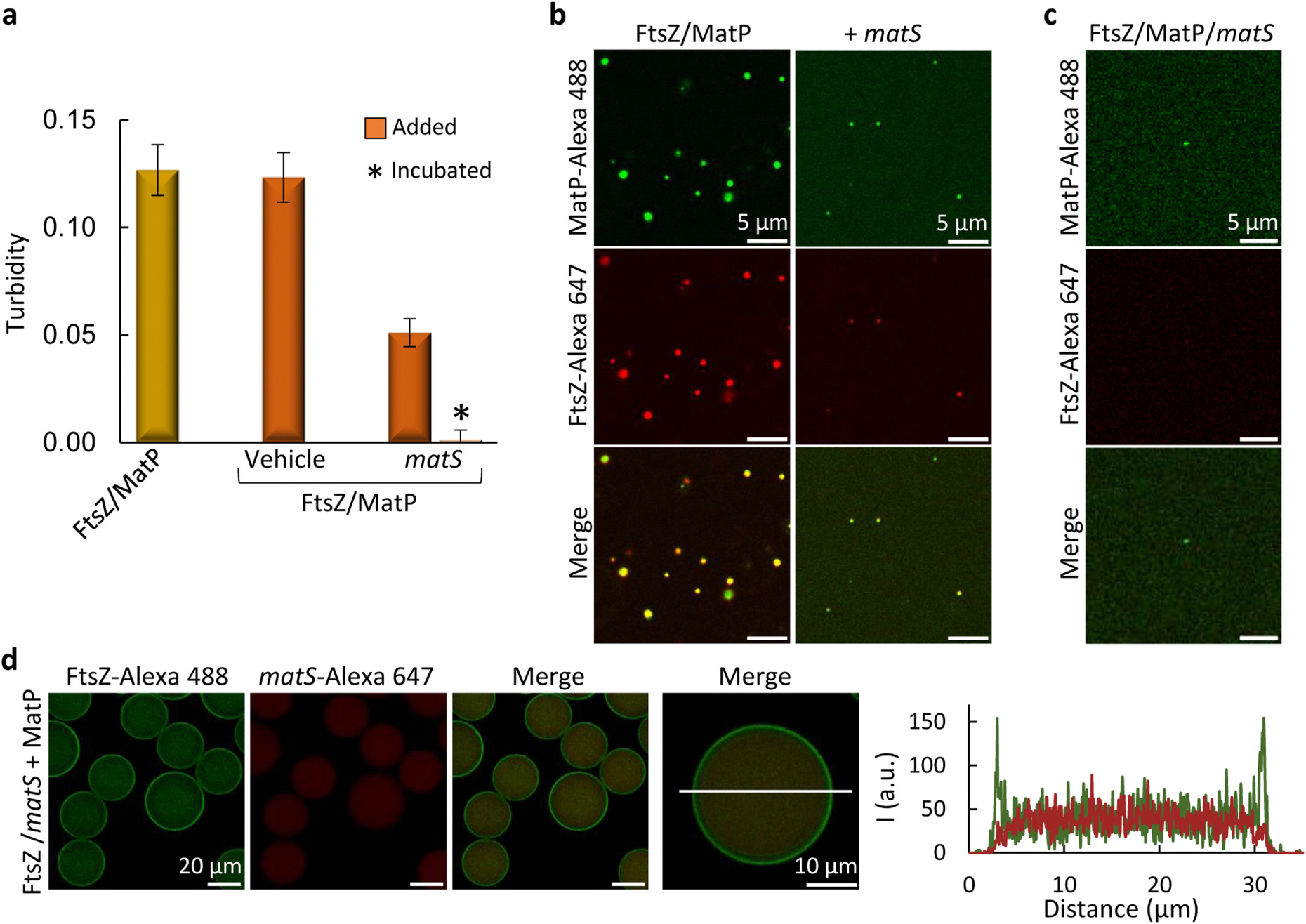
Detrimental effect of *matS* on FtsZ/MatP condensation. (a) Turbidity decrease of FtsZ/MatP solutions by *matS*, incubated with the protein from the beginning or added over preformed and 30 min-incubated condensates (measurements taken 10 min after addition). Dilution effects were discarded by addition of vehicle. Data are the average of three independent experiments ± SD. (b, c) Confocal images of FtsZ/MatP condensates before and after *matS* addition (b) and when FtsZ/MatP/*matS* were incubated from the beginning (c). (d) Microdroplets containing FtsZ, MatP and *matS,* showing lack of condensates. FtsZ/*matS* met MatP at the droplet formation junction. On the right, intensity profiles of the green and red channels along the line drawn in the image. Concentrations were 5 μM FtsZ, 3 μM MatP and 1 μM *matS* and labeled components. All experiments were performed under the *FtsZ/MatP-crowding conditions* (see Materials and methods).

We next asked whether the *matS* sequences specifically bound by MatP modulated the formation of FtsZ/MatP biomolecular condensates, in the same way or differently than that observed with the MatP homotypic ones. We found that, at 5:3:1 FtsZ:MatP:*matS* molar ratio, FtsZ/MatP condensates were disrupted by ds-oligonucleotides harboring a *matS* site, which largely reduced the associated turbidity, more efficiently when preincubated with the two proteins (Figs. 4a and S13a). Images also showed a considerable reduction in condensate abundance and size when *matS* was added over preformed condensates (Fig. 4b), and basically no condensates when *matS* was included in the FtsZ/MatP solutions from the beginning (Figs. 4c and S13b). As with MatP condensates, this inhibitory effect proved to be specific to double-stranded *matS*, as incorporation of ss-*matS* into the heterotypic condensates had no detectable effect on them (Fig. S13c). No condensates were observed upon co-encapsulation of *matS* with the two proteins, at the same molar ratio as above, in microdroplets (Fig. 4d). FtsZ was distributed between the membrane and the lumen, essentially as when encapsulated on its own, i.e. in the absence of MatP and *matS* (Fig. S12b), the latter localizing primarily in the lumen of the droplet. Hence, the specific DNA recognized by MatP inhibited the formation of FtsZ/MatP condensates both in bulk crowded solution and in cytomimetic platforms.

### 2.5. FtsZ and MatP directly interact and matS disrupts the complexes

Heterotypic FtsZ/MatP biomolecular condensation under conditions at which the individual proteins cannot phase separate strongly suggests an interaction between them, which would enhance multivalency rendering the system more prone to condensation. To test this hypothesis, we probed the interaction between FtsZ and MatP in *dilute solution buffer* (see Materials and methods) with 100 mM KCl by orthogonal biophysical methods.

**Fig. 5.**
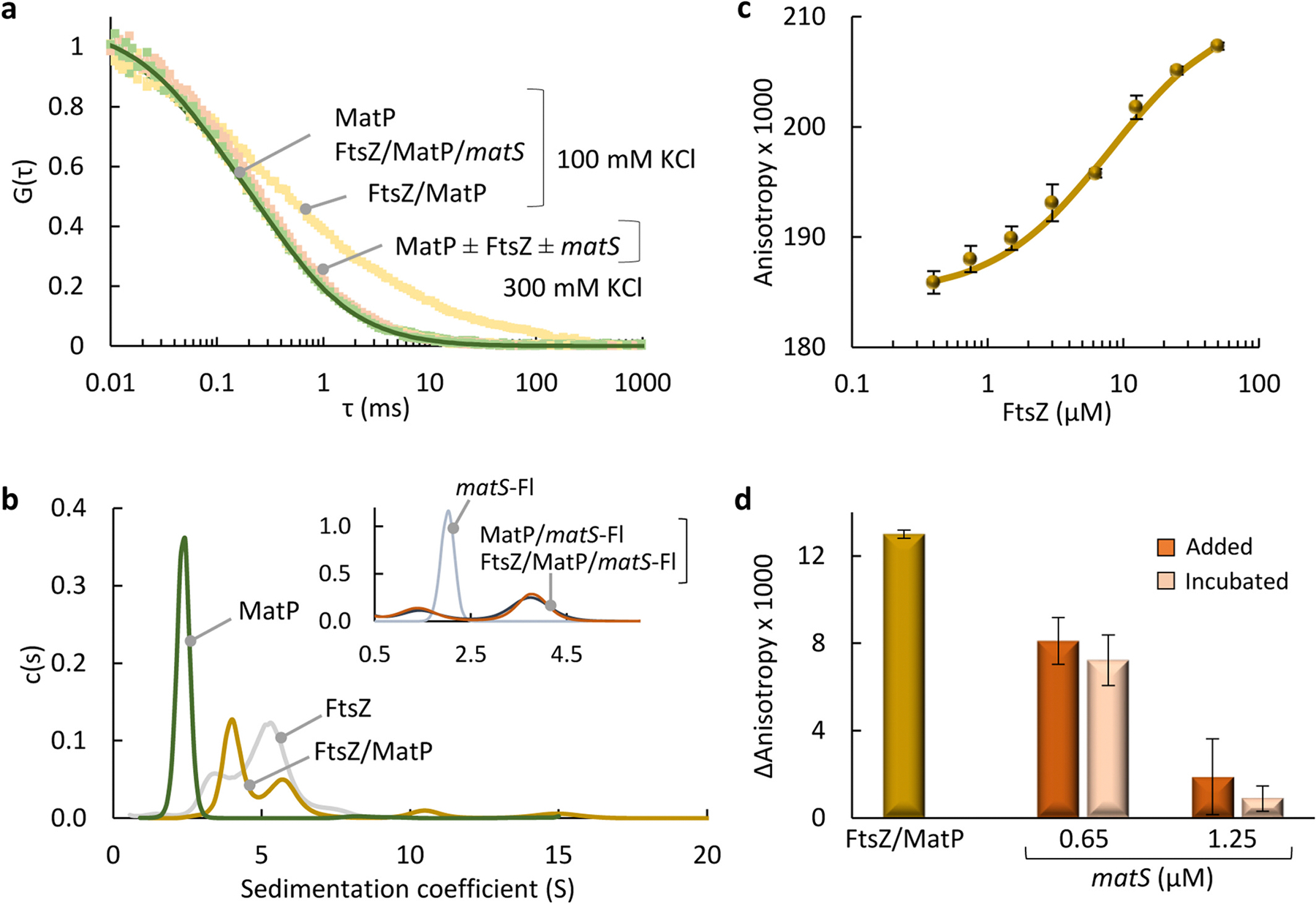
MatP binds to FtsZ through an interaction modulated by *matS*. (a) Normalized FCS autocorrelation curves of MatP (10 μM; 10 nM MatP-Alexa 488) and MatP with FtsZ (20 μM) in the presence and absence of *matS* (5 μM) at the specified KCl. Solid line corresponds to the fit of the model described in Materials and methods. (b) SV analysis of MatP and FtsZ, alone or combined, followed at 280 nm. Inset, profiles of *matS*-Fl with MatP or FtsZ/MatP followed at 493 nm. The distribution of *matS*-Fl alone is shown for comparison. Besides species remaining in solution, FtsZ/MatP samples contained a large fraction of higher order complexes fully sedimenting before reaching the assay final speed. Concentrations were 20 μM FtsZ, 7 μM MatP and 3.5 μM *matS*-Fl. (c) Fluorescence anisotropy binding titrations of MatP (50 nM MatP-Alexa 488, 96 nM total MatP) with FtsZ. Solid line is the fit of the model specified in Materials and methods and Supplementary material. (d) Fluorescence anisotropy change of FtsZ (5 μM, 50 nM FtsZ-Alexa 488) in the presence of MatP (2.5 μM) with and without *matS,* either incubated for 30 min with FtsZ/MatP or added over the preincubated proteins. Data in (c, d) are the average of three independent replicates ± SD. All experiments were performed in *dilute solution buffer* (see Materials and methods) with 100 mM KCl, unless otherwise stated.

FCS measurements of MatP, with MatP-Alexa 488 as tracer, showed autocorrelation curves (Fig. 5a) described with a single diffusion coefficient *D* (69 ± 2 μm^2^/s) close to that for a MatP dimer (66 μm^2^/s), calculated from the experimentally determined sedimentation coefficient, using Svedberg equation (s = 2.5 S; [26] and see below) and the theoretical mass (~35 kDa). Shifting of profiles to longer timescales upon addition of unlabeled FtsZ (Fig. 5a) was compatible with the formation of large FtsZ/MatP complexes, with some variability in the profiles even for replicates from the same sample. Incubation of the two proteins with *matS* (at 2:1 MatP:*matS* molar ratio) rendered autocorrelation profiles superimposable with those of solely MatP (Fig. 5a), meaning that *matS* inhibited the detected protein heterocomplexes. Increasing KCl to 300 mM resulted in overlapping profiles of MatP and FtsZ/MatP (Fig. 5a), indicating that large (or FCS-detectable) heterocomplexes were disfavored at this higher ionic strength. Therefore, FCS analysis revealed the formation of complexes between MatP and FtsZ involving electrostatic interactions and hindered by *matS.*

The interaction between both proteins and the effect of *matS* on the complexes were further analyzed by SV. As mentioned, MatP sedimented as a dimer (2.5 S; Fig. 5b and [26]), while FtsZ solutions contained a mixture of species (Fig. 5b) consistent with its tendency to form oligomers of variable size [43]. Sedimentation coefficient distributions of samples containing both proteins showed the complete disappearance of the peak corresponding to MatP, and the presence of two peaks partially overlapping with those of FtsZ alone, with a substantially lower intensity signal in the higher sedimentation peak (Fig. 5b). Multisignal analysis of profiles recorded at two wavelengths revealed that these peaks contained both MatP and FtsZ (Fig. S14a). We detected a large fraction (*~*50 %) of higher order complexes that fully sedimented before reaching the final speed of the assay. The effect of *matS* on the FtsZ/MatP complexes was analyzed by including fluorescein-labeled *matS* (*matS*-Fl) in the samples. Sedimentation coefficient distributions showed a major peak at 3.8 S, corresponding to MatP:*matS* complexes as previously described [26], and a minor amount of free *matS* (inset in Fig. 5b). This distribution was not modified in the presence of FtsZ (inset in Fig. 5b), indicating that all MatP was still available to form complexes with *matS* and ruling out the formation of FtsZ/MatP complexes, in good agreement with FCS results. Therefore, SV analysis confirmed the interaction between MatP and FtsZ, including the formation of large complexes, and the inhibition by *matS*.

The FtsZ/MatP complexes were evaluated by fluorescence anisotropy binding titrations of MatP (containing MatP-Alexa 488 as tracer) with increasing concentration of unlabeled FtsZ. Analysis of the concentration-dependent increase in the anisotropy associated with heterocomplex formation (Fig. 5c) rendered an apparent *K*_d_ of 8 ± 3 μM (or 18 ± 6 μM, considering intensity changes; see Materials and methods and Supplementary material for analysis details), representing the concentration of FtsZ yielding half of the maximum signal reached. Anisotropy was also used to evaluate the effect of *matS* on the interaction, in this case with FtsZ-Alexa 488 as tracer. Addition of MatP increased the anisotropy indicating complex formation (Figs. 5d and S14b), and incubation of FtsZ/MatP with *matS* or its addition over preformed complexes decreased the values (Fig. 5d), reflecting a concentration-dependent inhibition of the FtsZ/MatP interactions. Anisotropy was insensitive to the addition of MatP to labeled FtsZ when KCl was increased to 300 mM (Fig. S14b), a result compatible with a lack of interaction under these conditions. These experiments showed that *matS* was able to inhibit the formation of FtsZ/MatP complexes in solution and to dissociate those already formed.

Taken together, the orthogonal biophysical analyses confirmed the formation of heterocomplexes of moderate apparent affinity between FtsZ and MatP including large complexes, with electrostatic forces contributing to their formation and *matS* causing their disruption.

### 2.6. FtsZ within FtsZ/MatP biomolecular condensates reversibly forms GTP-triggered filaments decorated with MatP

**Fig. 6.**
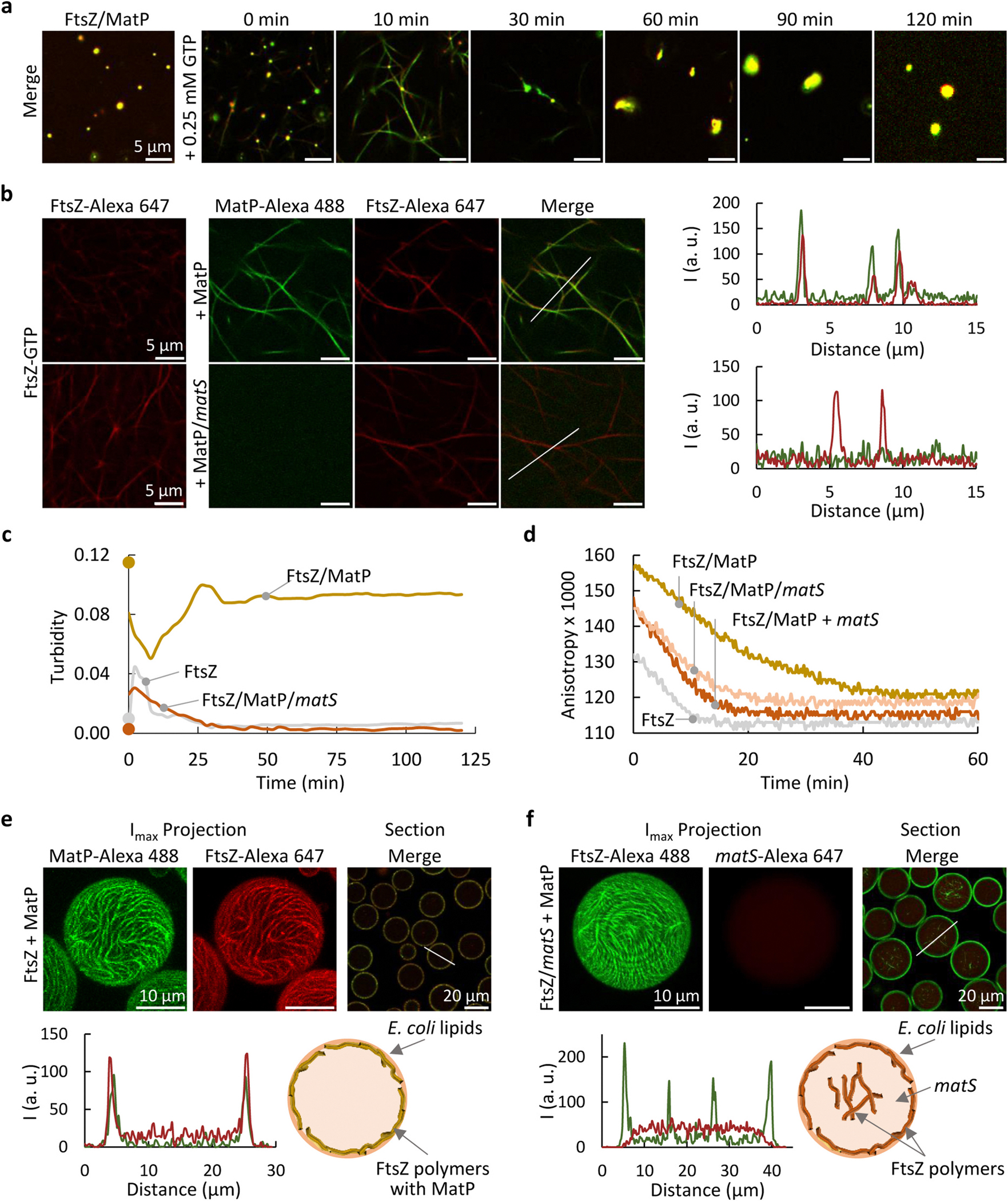
MatP decorates GTP-driven FtsZ bundles and dislodges in the presence of *matS*. (a) Merge confocal images of FtsZ/MatP condensates before and after triggering bundles containing FtsZ-Alexa 647 and MatP-Alexa 488 with GTP, and time-evolution showing condensate reassembly. Independent channels in Fig. S15. (b) Images of FtsZ bundles triggered by GTP before and after addition of MatP or MatP/*matS*. On the right, intensity profiles of the red and green channels along the lines depicted in the images, evidencing FtsZ/MatP colocalization in the polymers in the absence of *matS*. (c) Time evolution of FtsZ-GTP turbidity as compared to FtsZ-GTP/MatP with or without *matS*. Values prior to polymerization are represented as dots at 0 min. (d) Time evolution of fluorescence anisotropy of FtsZ-GTP (50 nM FtsZ-Alexa 488) with or without MatP or MatP/*matS* and effect of the addition of *matS* (+*matS*) to FtsZ/MatP immediately after polymerization. (e, f) Maximum intensity projections of the independent channels and merge equatorial sections of microdroplets containing FtsZ polymers and MatP without (e) and with *matS* (f). Intensity profiles of the green and red channels obtained along the line in the images, and schemes with the species distributions are also shown. MatP met FtsZ (e) or FtsZ/*matS* (f) at the droplet formation junction. All the experiments were performed in *FtsZ/MatP-crowding conditions* (see Materials and methods). When present, the concentrations of FtsZ, MatP and *matS* were 5, 3 and 1 μM, respectively. In (a, b, e and f) concentration of labeled components was 1 μM. Polymerization was induced with 0.25 mM (a, c, d) or 2 mM GTP (b, e, f).

GTP-induced assembly and GTP hydrolysis are hallmarks of FtsZ functionality retained in its previously described homotypic and heterotypic biomolecular condensates [37–39]. Analogously, GTP triggered bundles from FtsZ/MatP condensates, coexisting and interconnected with them (Figs. 6a and S15, *t* < 30 min). The bundles were initially weakly detected (*t* = 0), becoming visible shortly after (*t* = 10) in both the green (MatP-Alexa 488) and red (FtsZ-Alexa 647) channels, and signal colocalization strongly suggested that MatP incorporated into the FtsZ bundles. At this point, the number of condensates was significantly reduced while, as time passed, the polymers disbanded because of GDP accumulation due to GTP hydrolysis by FtsZ, and condensates where both proteins colocalized reassembled (Figs. 6a and S15, *t* > 30). Curiously enough, reassembled condensates seemed to be larger and less abundant than the ones from which polymerization was triggered (Figs. 6a and S15, cf. GDP and *t* = 60–120 min). Incorporation of MatP to FtsZ bundles was further confirmed through addition of MatP to preformed FtsZ polymers (Fig. 6b), allowing their inspection in the absence of condensates, and showing colocalization of the proteins. MatP seemed to prolong the lifetime of FtsZ polymers as, in its absence, no polymers could be detected 10 min after GTP addition (Fig. S16).

The response of FtsZ/MatP condensates to GTP was also monitored by turbidity (Fig. 6c) and fluorescence anisotropy (Fig. 6d). Turbidity decreased at short times after addition of GTP, consistent with the emergence of bundles and the concomitant dissociation of the condensates, as previously described for other FtsZ condensates [37,39]. Polymerization arising only from the protein outside the condensates would have increased the turbidity instead, because the overall signal would have been then contributed by both the intact condensates and the newly formed polymers. Turbidity reached a minimum at ~10 min, a time at which images showed mainly polymers (Figs. 6a and S15), and then increased, plateauing at ~30 min (Fig. 6c), a trend compatible with condensates reassembly (Figs. 6a and S15, t > 30 min). Without MatP, GTP induced the typical rapid increase in turbidity due to FtsZ bundles assembly, which did not form condensates, followed by a drop with polymers disassembly (Fig. 6c), the low signal precluding accurate determination of how long the polymers lasted. Anisotropy showed a time-dependent decrease in the value reached upon GTP addition to FtsZ/MatP condensates (with FtsZ-Alexa 488 as tracer) due to polymers dissociation [39], plateauing after ~40 min (Fig. 6d), in fair agreement with confocal and turbidity analysis. Values in the presence of MatP were higher during the whole time interval than in its absence, reflecting the presence of condensates (absent from the FtsZ only samples) and/or the formation of larger or more rigid polymers. Without MatP, anisotropy plateaued at ~10 min (Fig. 6d), further supporting shorter polymer duration than with MatP. Taken together, turbidity, confocal and anisotropy showed the evolution of FtsZ/MatP condensates towards polymers in the presence of GTP and their reassembly upon GTP exhaustion, and strongly suggested that MatP binds to FtsZ bundles retarding their disassembly.

We also studied the behavior of FtsZ polymers when co-encapsulated with MatP inside microdroplets with dextran as crowder and a lipid boundary. Polymerization was triggered by GTP just before encapsulation. Confocal images showed colocalization of FtsZ-Alexa 647 and MatP-Alexa 488 tracers at the membrane (Figs. 6e and S17a), forming polymers clearly observed in the maximum intensity projections and 3D reconstructions (Fig. 6e and Movie 1). Without MatP, FtsZ polymers were also found at the membrane, but their presence in the lumen was more evident than with MatP (Fig. S17b, and Movie 2), suggesting that the FtsZ/MatP interaction shifted them towards the membrane. Hence, MatP decorated FtsZ polymers also when reconstituted in microdroplets, increasing their tendency to locate at the lipid membrane.

### 2.7. matS sequences dissociate MatP from FtsZ bundles assembled in crowding conditions

The experiments described above showed that MatP accumulated at GTP-induced FtsZ bundles formed in crowding conditions, modifying their disassembly kinetics and distribution in cytomimetic systems. We asked if *matS* could have an effect on these observations, finding that its incubation with the proteins prevented incorporation of MatP into the polymers, its fluorescence signal appearing homogeneously distributed in the images (Fig. 6b, bottom). Likewise, addition of *matS* to FtsZ polymers decorated with MatP released the latter from them (Fig. S18), and MatP was not found on polymers triggered from FtsZ/MatP/*matS* samples (Fig. S19). Supporting these findings, turbidity profiles depicting the evolution of FtsZ/MatP/*matS* upon GTP addition were close to those of FtsZ alone (Fig. 6c), consistent with FtsZ/MatP condensates disruption (prior to GTP addition) and inhibition of MatP binding to the polymers by *matS*. Moreover, the notable difference between the anisotropy depolymerization profiles of FtsZ and FtsZ/MatP was largely reduced with *matS* either added to FtsZ/MatP polymers immediately after assembly or incubated with the proteins (Fig. 6d). Therefore, *matS* precluded incorporation of MatP to FtsZ polymers and was also able to detach it when already bound.

When *matS* was included in the FtsZ/MatP mixtures encapsulated in lipid-stabilized microdroplets with crowder in their interior, GTP-triggered FtsZ polymers were observed at the membrane and also in the lumen (Figs. 6f, S17c, S20 and Movie 3), adopting a distribution similar to that found in the absence of MatP (Fig. S17b). MatP was detected principally at the membrane (Fig. S20 and Movie 3), while *matS* remained homogeneously distributed in the lumen (Figs. 6f and S17c). Neither MatP nor *matS* colocalized with FtsZ polymers (Figs. 6f and S20). These experiments showed that the inhibition of MatP accumulation at FtsZ bundles by *matS* occurred also under cytomimetic conditions.

### 2.8. MatP binds GTP-induced polymers of FtsZ, and matS disrupts the complexes

The experiments above, performed in crowding conditions, suggested an interplay between MatP and GTP-triggered FtsZ bundles, consistent with an interaction. This prompted us to determine whether MatP could also bind the single-stranded protofilaments FtsZ forms in dilute solution [44,45]. FCS experiments with MatP-Alexa 488 as tracer showed a displacement of MatP profiles to longer times in the presence of FtsZ polymers (Fig. S21a) induced by GTP and stabilized by an enzymatic GTP regeneration system (RS, [46]), to keep them in solution during sufficient time to be characterized. SV analysis of samples containing the two proteins with GTP and RS showed the formation of very large complexes that sedimented in a large proportion before reaching the final speed of the assay, in contrast with the FtsZ polymers without MatP (Fig. S21b). Finally, MatP shifted the fluorescence anisotropy of FtsZ polymers (with FtsZ-Alexa 488 as tracer, Fig. S21c) to higher values, a result compatible with the formation of larger and/or more rigid species. In line with these results, electron microscopy imaging evidenced the presence of large aggregates formed when both proteins were present, in contrast with the single stranded polymers assembled by FtsZ on its own (Fig. S21d). GTPase measurements rendered similar values for FtsZ polymers in the absence and presence of MatP (5.5 ± 0.4 and 5.1 ± 0.2 mol of phosphate/mol FtsZ/min, respectively). Altogether, these experiments proved MatP binding to FtsZ polymers, forming complexes larger than the FtsZ polymers without MatP.

We also tested the effect of *matS* on MatP binding to FtsZ polymers and found it disrupted the complexes detected in the FCS experiments, leading to profiles approximately matching those of samples containing only MatP, the labeled element in these assays (Fig. S21a). Analogously, sedimentation coefficient distributions of FtsZ polymers were roughly the same as those of FtsZ with MatP/*matS* incubated before triggering assembly with GTP (Fig. S21b). Moreover, anisotropy experiments showed a concentration-dependent antagonistic effect of *matS*, whether added prior to or after triggering polymerization of the FtsZ/MatP mixtures. Thus, the anisotropy values of FtsZ-GTP/MatP gradually approached those of FtsZ polymers without MatP as the concentration of *matS* in the samples increased (Fig. S21c). Therefore, *matS* blocked MatP binding to FtsZ protofilaments in a concentration-dependent manner.

## 3. Discussion

**Fig. 7.**
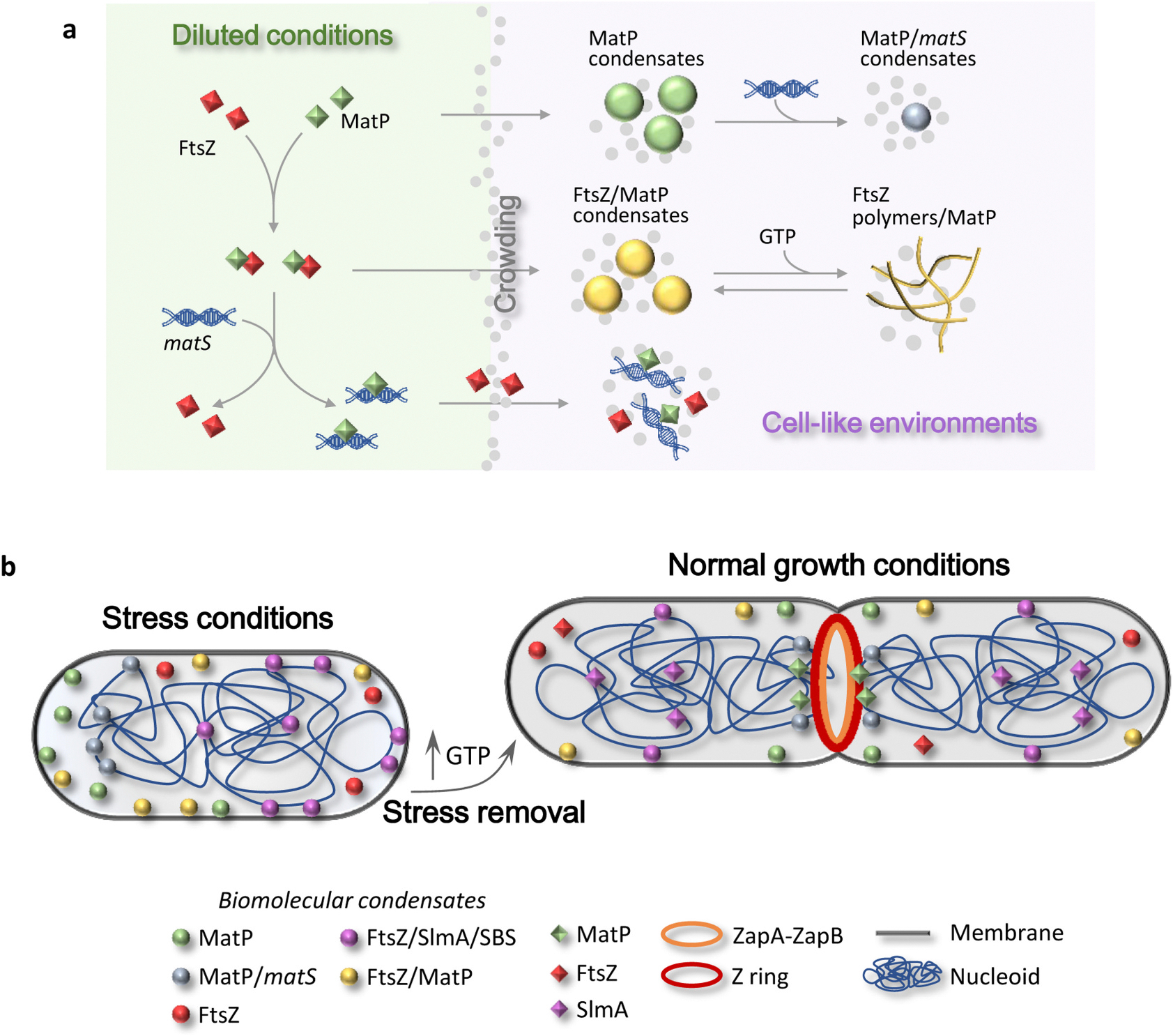
Schematic representation of the assembly and regulation of MatP biomolecular condensates and hypothetical role in cells under stress conditions. (a) Top: MatP forms crowding-driven condensates (center) where *matS* can incorporate (right). Middle: MatP interacts with FtsZ (left) and forms heterotypic condensates in crowding conditions (center) that interconvert with MatP-bound FtsZ polymers in response to GTP addition and depletion (right). Bottom: *matS* disrupts the FtsZ/MatP complexes (left), hindering condensation (right). (b) Under stress conditions, MatP could be transiently stored within condensates, preferentially at the membrane, and reduced GTP levels [40,41] would favor FtsZ storage in homotypic [37] and heterotypic condensates (with MatP or SlmA [38]), which would facilitate halting MatP and FtsZ-dependent cell cycle events to cope with stress. MatP condensates with *matS* might contribute to nucleoid compaction, a known role of MatP [22], possibly conferring additional resistance, as described for other NAPs [6]. Upon stress removal, an increase in cellular GTP concentration would trigger, from FtsZ/MatP condensates, FtsZ polymers with bound MatP, which would be dissociated by *matS*. Under normal growth conditions, condensates may be found to a different extent. MatP condensates may still remain at the membrane to temporally sequester MatP from the chromosome, facilitating binding of other nucleoid binding factors to regions that would be occluded by MatP when bound to the chromosome. Under division conditions, condensates containing FtsZ would also serve to store the large amount of this protein outside the division ring [45,47], or the whole FtsZ pool under non-division conditions. MatP condensates at the membrane would be regulated by *matS* sites, which would displace MatP to the cytoplasm/nucleoid when required. MatP condensates enriched in *matS* could facilitate the role of this protein in Ter macrodomain compaction.

Here, we show that the bacterial protein MatP, which participates in FtsZ ring positioning, DNA organization and coordination of cell division with chromosome segregation, undergoes biomolecular condensation in crowding cytomimetic conditions (Figs. 7a and S22). These novel assemblies are negatively regulated by *matS* DNA sites that partition into them and, when formed at the microdroplets membrane for which the protein displays affinity, partially shifts the condensates towards the lumen. Accumulation of FtsZ into MatP condensates suggests a direct interaction between these two proteins, confirmed by biophysical analysis in dilute solution, that facilitates phase separation through heterotypic condensation.

MatP phase separation is likely driven by multivalency, as it contains multiple binding domains: a central ribbon-helix-helix mediating dimerization and interaction with *matS* sites; an N-terminal four-helix bundle also interacting with DNA; and a C-terminal flexible coiled-coil domain important for protein-protein recognition [24,25]. In this regard, recent coarse-grained molecular simulations show the presence of coiled-coil domains suffices to support biomolecular condensation [48], and many prokaryotic and eukaryotic proteins containing such domains phase separate (e.g. the cell polarity protein PodJ from *C. crescentus* [49], centrosomal proteins, transcription factors and RNA binding proteins ([48] and references therein)).

MatP condensation is consistent with its originally described uneven cellular distribution pattern in bacteria [22]. The protein accumulates at specific locations depending on the cell cycle stage, forming discrete foci that shift from the new cell pole to the cell center, ultimately splitting into two sister foci that remain juxtaposed until they move away from midcell. MatP foci have been reported in other in vivo studies, and their number and distribution analyzed under different conditions [24–28]. Dynamic and reversible MatP biomolecular condensates such as those we have identified in cytomimetic systems and their refined control by *matS* could provide a mechanism for rapid mobilization and reorganization of the MatP pool within the cell. MatP condensates allowing subsequent incorporation of *matS* sequences, or even assembled alongside low *matS* concentrations, might also aid in the DNA structural arrangement mediated by this protein, in line with the earlier described reorganization of chromatin by nuclear condensates [50]. The fact that these condensates are favored by macromolecular crowding would facilitate their assembly near the chromosome, which is coated by a high density of nucleoid associated proteins (NAPs), covering ~30 % of the genome in *E. coli* [51]. Crowding plays a role in the phase separation of NAPs such as histone-like heat-unstable nucleoid protein (HU), DNA-binding protein from starved cells (Dps), single-stranded DNA binding protein (SSB) and RNA polymerase (RNAP) [52–55].

Interestingly, the mechanisms regulating MatP condensates are strikingly reminiscent of those reported for SSB, including sequestration of DNA at low concentrations compared with those of accompanying proteins, and inhibition of phase separation at higher DNA concentrations [54]. Furthermore, in both cases condensates assemble at the membrane with which the proteins interact. SSB condensates serve to store this protein alongside interacting molecules, allowing rapid mobilization when required to repair damaged DNA. In the case of MatP, its temporal removal from the chromosome and accumulation at the membrane have been proposed to facilitate the function of proteins that help segregate the Ter region, like FtsK [26], and perhaps Topoisomerase IV [35]. Condensates containing solely MatP would be detached from the membrane upon incorporation of *matS,* and high specific DNA concentrations that might locally arise would antagonize their assembly. Condensates of SSB, as well as those of Dps and HU, aggresomes, ribonucleoprotein bodies or polyP granules have been connected to stress resistance [6], conferred through DNA compaction in some instances [52,53]. MatP phase separation may have similar implications, as it is known to constrain the Ter macrodomain [56].

Our results show direct interaction between MatP and FtsZ that strongly promotes phase separation into compartments in crowding cytomimetic systems (Figs. 7 and S22). These compartments could be regarded as coacervates formed by a positively and a negatively charged proteins (MatP, pI 9.5; FtsZ, pI 4.6), consistent with their strong dependence on ionic strength. FtsZ/MatP heterocomplexes were also detected in dilute conditions, including large complexes that could potentially represent incipient biomolecular condensates. Hypothetically, these condensates could provide storage for FtsZ in non-dividing cells or accommodate the ~60 % cellular FtsZ that is not in the FtsZ ring [45,47]. MatP and FtsZ stored within these reservoirs, located principally at the membrane according to our experiments in cytomimetic systems, could be easily mobilized. Besides the negative regulation by *matS,* FtsZ/MatP condensates are also susceptible to disassembly by GTP, which triggers FtsZ polymers with bound MatP, ultimately dissociated from them by *matS* (Figs. 7a and S22). This additional pathway is particularly interesting for preformed MatP condensates, which are more resistant to the action of *matS*. It remains to be determined whether FtsZ/MatP dissociation is required for canonical interaction of each protein with ZapA and ZapB, respectively, or if the FtsZ/MatP interaction itself is part of the Ter-linkage.

A hallmark of quiescent states is reversible arrest of cell growth and division [57], for which storage of cytokinesis factors and regulators like MatP and FtsZ inside DNA- and GTP-responsive compartments might be an effective strategy (Fig. 7b). This was previously proposed for condensates of FtsZ, alone or with the nucleoid occlusion factor SlmA [37,38]. Interestingly, DNA binding strongly promotes FtsZ/SlmA phase separation [38], giving rise to condensates in which FtsZ and its inhibitor are sequestered. Such a mechanism might be expected to inhibit FtsZ ring assembly even in the presence of agonists like ZapA, also recruited to the condensates where it is neutralized by SlmA [39]. In contrast, depending on the local concentration, *matS* binding is detrimental for FtsZ/MatP condensate formation, perhaps facilitating Ter-linkage assembly and normal cytokinesis upon stress release. The other key player in the regulation of FtsZ-containing condensates is GTP, which is strongly depleted in quiescent states [40,41], favoring the condensation-prone oligomeric forms of FtsZ (Fig. 7b). Once the stress is over, an increase in GTP concentration would disassemble these structures allowing normal cell division.

Our work reinforces the idea that bacterial proteins from *E. coli* playing key roles in the regulation of the cell cycle display the ability to assemble biomolecular condensates under crowding cytomimetic conditions. As we show here, these compartments are sensitive to conditions such as ionic strength and to specific ligands like nucleic acids, lipid membranes or nucleotides, determining their formation, composition, localization and interconversion with other structures, which may ultimately impact the function of the proteins involved. These features may be also of interest in the design of programmable condensates to tune cell function or to design synthetic cells displaying dynamic reversible compartments, following encapsulation approaches like the ones used here. Besides, condensates containing bacterial cell cycle proteins could potentially be involved in the development of persisters and, hence, could be regarded as new targets for molecules that can kill pernicious bacteria.

## 4. Materials and methods

Detailed information about reagents, confocal microscopy and anisotropy can be found in the Supplementary material.

### 4.1. Protein purification and labeling, and DNA hybridization

FtsZ and MatP were purified as previously described (FtsZ, calcium-induced precipitation method [43]; untagged MatP [26]), and stored in aliquots at −80 °C. For the experiments, MatP was equilibrated in 50 mM Tris-HCl, pH 7.5 and 300 mM KCl to avoid protein aggregation, and conditions subsequently adjusted to those conditions required for each experiment. FtsZ was dialyzed in 50 mM Tris-HCl, pH 7.5, with 5 mM MgCl_2_ and the specified KCl concentration. Proteins were labeled at their amino groups with Alexa Fluor 488 or Alexa Fluor 647 carboxylic acid succinimidyl ester dyes as described [26,58]. Briefly, FtsZ was labeled after triggering polymerization with GTP, to minimize interference of the dye with protein assembly. MatP was dialyzed against 50 mM HEPES, pH 7.5, 300 mM KCl, 1 mM EDTA, 10 % glycerol buffer and allowed to react at room temperature with Alexa Fluor 488 (30 min, 2-fold molar excess of dye) or with Alexa Fluor 647 carboxylic acid succinimidyl ester (20 min, 1.5-fold molar excess of dye), and the unreacted dye separated from the labeled protein by using a HiTrap desalting column. The labeling ratios, calculated from the molar absorption coefficients of proteins and dyes, were always <1 mol of dye per mole of protein. Single stranded oligonucleotides containing the *matS19* sequence recognized by MatP [22] or a nonspecific sequence of similar length, either unlabeled or labeled with Alexa Fluor 647 or fluorescein in the 5′ end, were hybridized with the complementary oligonucleotides as described [26].

### 4.2. Preparation of samples in crowding bulk solution

Crowding bulk solutions were prepared in Protein LoBind^®^ tubes (Eppendorf) by adding the protein(s) and GTP, when required, to a solution containing the specified crowder, dextran 500 or Ficoll 70, previously dialyzed in 50 mM Tris-HCl, pH 7.5 without or with 100 mM or 300 mM KCl. The final buffer conditions were adjusted to be 50 mM Tris-HCl, pH 7.5, 5 mM MgCl_2_, 200 g/L dextran and 100 mM KCl (*MatP-crowding conditions*) for most analysis of MatP condensates, and 50 mM Tris-HCl, pH 7.5, 1 mM MgCl_2_, 150 g/L dextran and 200 mM KCl (*FtsZ/MatP-crowding conditions*), for analysis of FtsZ/MatP condensates, unless otherwise stated.

### 4.3. Turbidity assays and determination of c_sat_

Turbidity measurements at 350 nm were performed using a Varioskan Flash Plate reader (Thermo Fisher Scientific, MA, USA) following a protocol described elsewhere [37] but with 85 μL samples in 384-well clear polystyrene, flat bottom microplates (Greiner Bio-One). Salt shift experiments were conducted by adding a small volume of a KCl solution into the condensates samples. Vehicle refers to controls where an equivalent volume of buffer was added instead, accounting for possible dilution effects. Data collection started after 30 min incubation at room temperature or, in samples monitored with time, immediately after addition of the studied element (*matS*, GTP, etc.). Results are the average ± SD or representative profiles from at least three independent experiments. The concentration threshold for condensate formation, *c*_sat_, was determined by fitting a linear model to the data scaling with the protein concentration using user-written scripts and functions in MATLAB (ver. 7.10; MathWorks, Natick, MA) as described earlier [37,59], and corresponds to the x-intercept. Error in *c*_sat_ was obtained by error propagation from the errors of the slope and the y-intercept representing confidence limits at 68 %.

### 4.4. Microfluidics encapsulation

Encapsulation, in microfluidic devices produced by conventional soft lithography [60], involved mixing two aqueous streams in a 1:1 ratio just before the droplet formation junction. For multiple elements, aqueous phases of various compositions were assayed accounting for possible effects from incubation times: *matS*/MatP and FtsZ/MatP, including the two elements together in both aqueous phases or each one in a different solution with analogous results; FtsZ/MatP with *matS*, the latter included either in the stream with FtsZ or with MatP, with similar results. FtsZ polymerization was induced just before encapsulation, adding GTP in the Fts*Z*-free aqueous solution. A third stream contained mineral oil with the *E. coli* lipid mixture (20 g/L), prepared as described earlier [26]. Solutions were delivered at constant flows of 150 μL/h (oil phase) and 10 μL/h (aqueous phases) by automated syringe pumps (Cetoni GmbH, Germany), and production in the microfluidic device was monitored with an Axiovert 135 fluorescence microscope (Zeiss). Microdroplets were collected and observed 30 min after production.

### 4.5. Confocal microscopy

Samples, either crowding bulk solutions or microfluidics droplets, were visualized using a Leica TCS SP5 inverted confocal microscope as described [39]. Condensates were incubated for 30 min before visualization. For each sample, several images were registered. Production of images and data analysis was done with ImageJ [61]. In a group of images corresponding to the two different channels and the merge of a particular field, a relevant scale bar is specified only in one image for simplicity. Intensity profiles, maximum intensity projections and 3D reconstructions, always corresponding to the raw images, were obtained with the straight-line, Z-projection and 3D-projection tools, respectively. Condensates diameter distributions were obtained with the particle analysis option as described [37] and represented as violin plots using Origin 2024b. Statistical analysis was performed with GraphPad 10.4.1 software by ANOVA on ranks test (Kruskal-Wallis) followed by Dunn’s test. Brightness was uniformly increased in the whole image, only in the specified cases, using Microsoft PowerPoint. Representative images of at least three individual experiments are shown, except for microfluidic encapsulations that were repeated at least twice.

### 4.6. Fluorescence correlation spectroscopy (FCS)

FCS measurements were conducted on a Microtime 200 (PicoQuant) time-resolved confocal fluorescence microscope equipped with a pulsed laser diode head (LDH-P-C-485) for excitation, as previously described [39]. Experiments were done in *dilute solution buffer*: 50 mM Tris-HCl, pH 7.5, 5 mM MgCl_2_ and 100 or 300 mM KCl, as specified. For FCS measurements, 0.05 g/L BSA was added, to avoid nonspecific adsorption to the surfaces, also prevented by using pegylated coverslips. When specified, GTP and an enzymatic GTP regeneration system (RS) were included. Shown autocorrelation curves are representative of, at least, three independent experiments (5 profiles each). Analysis was performed with the FFS Data Processor Software (version 2.4 extended, SSTC [62]), using models with a term for the triplet state dynamics and one-diffusing species for the MatP or FtsZ/MatP/*matS* curves. Reported diffusion coefficients are the average of three independent measurements ± SD. Quantitative analysis was omitted for FtsZ/MatP in view of the multiplicity of species present, including very large complexes.

### 4.7. Sedimentation velocity assays (SV)

Samples, in *dilute solution buffer* with 100 mM KCl, were centrifuged at 48,000 rpm in an Optima XL-I analytical ultracentrifuge (Beckman-Coulter Inc.) as earlier described [39], and profiles were recorded by absorbance at 250 or 280 nm, or at 280 and 493 nm when including *matS*-Fl. When specified, GTP and the aforementioned GTP RS were included, and profiles recorded by Raleigh interference. Experiments were repeated at least twice. Differential sedimentation coefficient distributions were calculated by SEDFIT [63], and heterocomplex formation was qualitatively characterized by multi-signal sedimentation velocity (MSSV) with SEDPHAT [64].

### 4.8. Fluorescence anisotropy

Anisotropy experiments were performed in a Spark^®^ Multimode microplate reader (Tecan) as described [39]. FtsZ/MatP binding isotherms were obtained using 50 nM MatP-Alexa 488 as tracer, in the same buffer used for the FCS experiments, with 100 mM KCl. Binding analysis was conducted with a simple 1:1 model using BIOEQS software [65,66], to obtain apparent *K*_d_ corresponding to the FtsZ concentration rendering half of maximum signal reached. Uncertainties in the parameters retrieved were calculated with the same software by rigorous confidence limit testing at the 67 % level, and error propagation. The effect of *matS* on the FtsZ/MatP interaction and titrations of FtsZ with MatP were conducted in *dilute solution buffer* with 100 or 300 mM KCl, as specified. Anisotropy of FtsZ polymers with or without MatP and/or *matS* was monitored with time, after GTP addition in *FtsZ/MatP-crowding conditions* or measured at time 0 in *dilute solution buffer* with 100 mM KCl. Reported anisotropy data are the average ± SD or representative profiles from, at least, three independent experiments.

### 4.9. GTPase activity measurements

FtsZ GTPase activity was determined using BIOMOL^®^ GREEN assay (Enzo Life Sciences), essentially as described in [67]. Samples contained 10 μM FtsZ and, when present, 5 μM MatP in *dilute solution buffer* with 100 mM KCl. Reported data are the average of three independent experiments ± SD.

### 4.10. Electron microscopy

Electron microscopy images were acquired under low dose conditions using a Thermo Fisher TALOS L120C transmission electron microscope operated at 120 kV and equipped with a Thermo Fisher CETA-F camera. 5 μM FtsZ, with or without 2.5 μM MatP, was incubated with 2 mM GTP in *dilute solution buffer* with 100 mM KCl for 5 min, adsorbed to glow-discharged carbon coated grids and stained with 2 % uranyl acetate. Representative images of 2 individual experiments are shown.

## Supplementary Material

supplemental movie 3

supplemental movie 2

supplemental movie 1

Supplementary file

Supplementary data to this article can be found online at https://doi.org/10.1016/j.ijbiomac.2025.142691.

## Data Availability

All data are available from the corresponding authors upon request.
